# Toxicological Evaluation of the Roots of *Ficus pandurata* Hance var. *angustifolia* Cheng

**DOI:** 10.1002/fsn3.71979

**Published:** 2026-06-02

**Authors:** Yamin Chen, Zhijing Fu, Fangfang Ma, Siyuan Wang, Junjie Pan, Huaxing Luo, Zhen Liu, Jianguo Chen, Jingjin Hu, Yangjunna Zhang, Kejun Cheng, Ye Jin, Xuefeng Qu

**Affiliations:** ^1^ China National Center for Food Safety Risk Assessment Beijing P. R. China; ^2^ School of Pharmacy, School of Food Science and Engineering Hangzhou Medical College Hangzhou Zhejiang P. R. China; ^3^ Agriculture and Rural Bureau of Suichang County Lishui Zhejiang P. R. China; ^4^ Lishui Institute of Agriculture and Forestry Sciences Lishui Zhejiang P. R. China; ^5^ College of Agriculture and Biotechnology Lishui University Lishui Zhejiang P. R. China

**Keywords:** acute oral toxicity test, development toxicity, genotoxicity, root of *Ficus pandurata* Hance var. *angustifolia* Cheng, subchronic toxicity

## Abstract

This study aimed to systematically evaluate the safety of roots of *Ficus pandurata* Hance var. *angustifolia* Cheng (RFPH) using comprehensive cellular and animal toxicological assays. Two‐year‐old RFPH roots were processed into an aqueous extract powder, with 1 g of extract equivalent to 30 g of raw material. Acute oral toxicity, bacterial reverse mutation, chromosome aberration, mammalian micronucleus, teratogenicity, and 90‐day repeated‐dose oral toxicity tests were conducted in SD rats and ICR mice. No significant signs of toxicity or mortality were observed in mice or rats administered 10.0 g/kg BW of RFPH aqueous extract powder in the acute toxicity test. The bacterial reverse mutation, mammalian micronucleus, and chromosome aberration tests were negative, suggesting no mutagenic potential under the experimental conditions. In the 90‐day repeated‐dose oral toxicity study, the NOAELs were 2.72 g/kg BW/day for female rats and 2.51 g/kg BW/day for male rats, corresponding to 81.6 and 75.3 g/kg BW/day of raw RFPH, respectively. The study design and key findings are summarized in the graphical abstract. These results provide toxicological safety evidence supporting the potential application of RFPH in food, functional food, and herbal product development.

## Graphical abstract

Toxicological evaluation of the roots of *Ficus pandurata* Hance var. *angustifolia* Cheng for safe utilization. Two‐year‐old RFPH roots were prepared as an aqueous extract powder, with 1 g equivalent to 30 g of raw material. Acute oral toxicity, genotoxicity, teratogenicity, and 90‐day repeated‐dose oral toxicity tests were performed in SD rats and ICR mice. RFPH showed no apparent acute toxicity at 10.0 g/kg BW, negative genotoxicity results, and no treatment‐related adverse effects in the 90‐day study, with NOAELs of 2.72 g/kg BW/day in female rats and 2.51 g/kg BW/day in male rats.

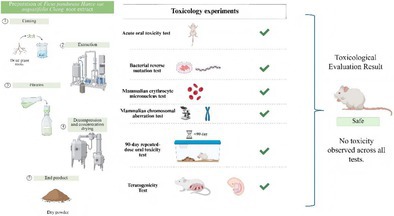

## 1 Introduction

*Ficus pandurata* Hance var. *angustifolia* Cheng is a variant of *Ficus pandurata* Hance, belonging to the genus *Ficus* in the Moraceae family (Chen et al. 2020). This plant is primarily distributed across southeastern China, including provinces such as Zhejiang, Guangdong, Hainan, Guangxi, Fujian, and Hunan (Editorial Committee of Flora Republicae Popularis Sinicae, Chinese Academy of Sciences 2004). The roots of *Ficus pandurata* Hance var. *angustifolia* Cheng (RFPH) are frequently utilized as a primary ingredient in medicinal diets and as a seasoning, particularly in soup preparation. RFPH could provide various health benefits, including enhancing gastric function, facilitating digestion, alleviating rheumatism, and protecting intestinal barrier integrity, as well as cognitive function (Zhejiang Food and Drug Administration 2016; Zhang et al. 2023). In the Zhejiang region of China, it is commonly used in folk customs to prepare soup, serving as a daily health maintenance practice.

The key nutritional components of RFPH include crude protein, crude fat, dietary fiber, amino acids, and minerals (Ying et al. 2012). The RFPH is rich in amino acids, with a total amino acid content of 2.90 g/100 g, including all six essential amino acids (Thr, Val, Ile, Leu, Phe, Lys) (Ying et al. 2012). The RFPH is rich in flavonoids, with a total flavonoid content of 12.74 g/kg (Ying et al. 2012). Flavonoids possess a wide range of biological functions, including antioxidant, anti‐inflammatory, antidiabetic, cardiovascular‐protective, and anticarcinogenic properties (Dias et al. 2021; Serafini et al. 2010; Ghorbani 2017; Alkhatib et al. 2017). Recent studies indicate that the flavonoid components of *Ficus pandurata* Hance var. *angustifolia* Cheng improve colonic pathological damage and abnormal intestinal microbiota structure in mice with circadian rhythm disorders (Liu et al. 2019; Zhang et al. 2023). The RFPH is rich in flavonoids, which suggests that its health‐related functions are worthy of further investigation.

However, the lack of comprehensive toxicity data for RFPH may obscure critical issues, such as a narrow therapeutic window or an unfavorable efficacy‐toxicity ratio. Therefore, it is essential to conduct toxicological evaluations, including cytotoxicity assays and animal behavioral observations, to establish safety thresholds and provide robust toxicological evidence for its development as a new food ingredient and functional food.

## 2 Materials and Methods

### 2.1 Material

**FIGURE 1:**
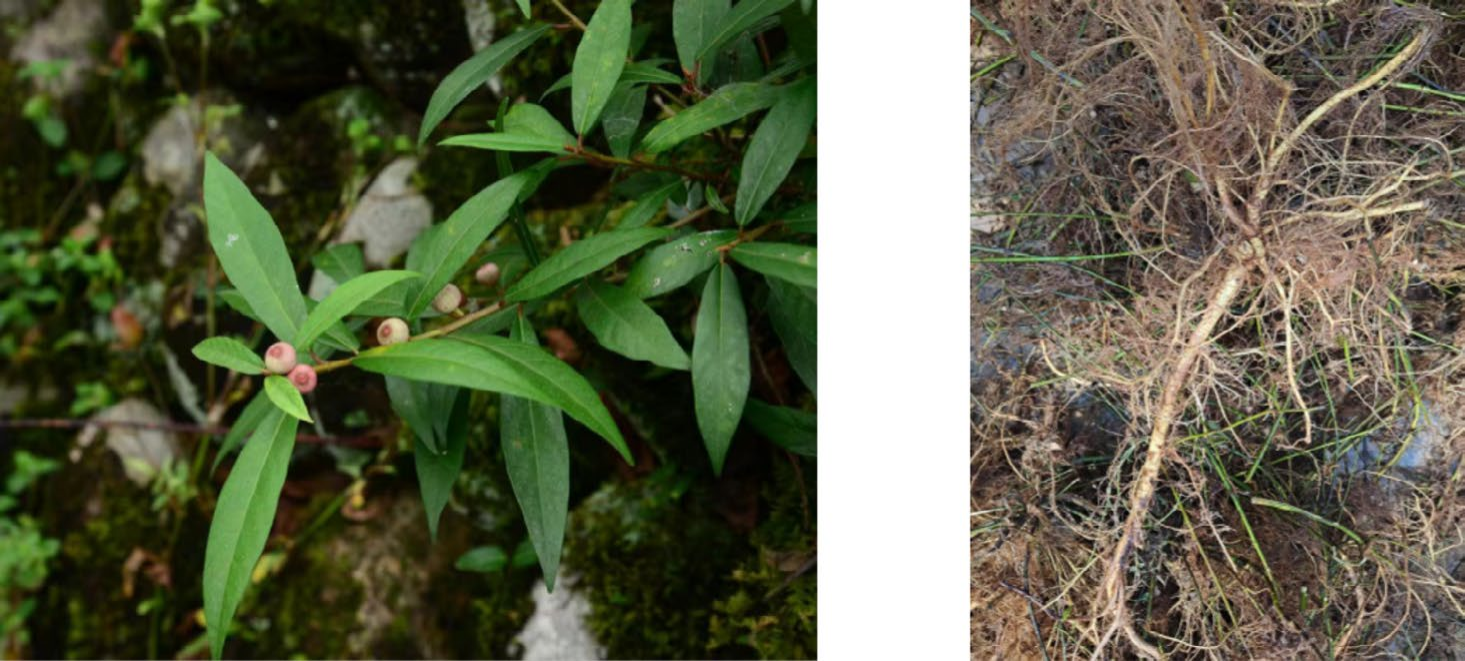
Images of *Ficus pandurata* Hance var. *angustifolia* Cheng and root of *Ficus pandurata* Hance var. *angustifolia* Cheng (Cheng et al. 2025).

Two‐year‐old *Ficus pandurata* Hance var. *angustifolia* Cheng plants were selected from the standardized cultivation base in Suichang County, Lishui City, Zhejiang Province, China, and the RFPH were harvested in April (Figure 1). The *Ficus pandurata* Hance var. *angustifolia* Cheng is artificially cultivated. The plant material was authenticated by Professor Kejun Cheng from Lishui Institute of Agriculture and Forestry Sciences as *Ficus pandurata* Hance var. *angustifolia* Cheng. Morphologically, RFPH is long and cylindrical with branched structures; the surface ranges from green to brown and is hard in texture, and the xylem on fracture is yellowish white. The spray‐dried powder of *Ficus pandurata* Hance var. *angustifolia* Cheng root was produced by Zhejiang Suichang Limin Pharmaceutical Co. Ltd. and prepared as follows: fresh roots (720 kg) were subjected to heat‐reflux extraction twice, each for 2 h. The two aqueous extracts were combined and concentrated at 65°C–75°C to a relative density of approximately 1.1. The resulting concentrated extract was then spray‐dried under controlled conditions: inlet air temperature 170°C–190°C, outlet air temperature 70°C–90°C. Finally 24 kg of spray‐dried powder of *Ficus pandurata* Hance var. *angustifolia* Cheng root was obtained for subsequent experiments.

### 2.2 Animals

SPF‐grade ICR mice and SD rats were used in this study. Animals were equally divided by gender for toxicological evaluations, including acute oral toxicity, micronucleus assays, chromosome aberrations, and a 90‐day repeated‐dose oral toxicity study. Prior to experiments, all animals acclimatized for 72 h in a controlled facility (20°C–25°C, 40%–70% humidity, 12‐h light–dark cycle).

This study followed the “Guidelines for Ethical Review of Animal Welfare in Experimental Animals” (GB/T 35892‐2018) and was approved by the Animal Care and Use Committee of Hangzhou Medical College (approval no. ZJCLA‐IACUC20100008). The tests adhered to relevant Chinese national standards: acute oral toxicity (GB 15193.3‐2014), bacterial reverse mutation (GB 15193.4‐2014), mammalian erythrocyte micronucleus (GB 15193.5‐2014), spermatogonial chromosome aberration (GB 15193.8‐2014), 90‐day repeated‐dose oral toxicity (GB 15193.13–2015), and teratogenicity (GB 15193.14‐2015).

### 2.3 Acute Oral Toxicity Test

Acute oral toxicity testing helps identify potential adverse reactions to substances and provides evidence for subsequent toxicity studies (Walum 1998). The ICR mouse model allows for determining toxic dose thresholds, while mammalian micronucleus and chromosome aberration assays assess genetic material damage (Zeiger 1998). A 90‐day subchronic oral toxicity study in SD rats further evaluates cumulative toxic effects on organ systems. Using ICR mice and SD rats establishes a basis for dose selection in further studies.

Ten healthy adult ICR mice (18–22 g) and 10 SD rats (180–220 g), equally divided by gender, were used in the study. Mice and rats were orally administered 10.0 g/kg body weight (BW) of RFPH water extract dry powder (equivalent to 300 g/kg BW of RFPH) for acute oral toxicity evaluation (GB 15193.3‐2014). The dry powder (100 g) was dispersed in 200 mL of 1% carboxymethyl cellulose sodium (CMC‐Na) solution. ICR mice were fasted for 6 h before a single 20 mL/kg BW gavage, followed by feeding 2 h post‐administration. SD rats were fasted for 16 h before two oral administrations of 10 mL/kg BW at 3‐h intervals, with feeding resumed 3 h post‐administration.

After gavage, a 14‐day observation period was conducted, monitoring behavior, toxicity signs, survival, and body weight changes. At the end, all animals were euthanized, and gross pathological examinations were performed, documenting any macroscopic changes for further toxicological studies.

### 2.4 Bacterial Reverse Mutation Test

Histidine auxotrophic *Salmonella typhimurium* strains (TA97a, TA98, TA100, TA102, TA1535; MOLTOX) were used for the bacterial reverse mutation test. Prior to use, the strains were characterized to ensure genetic stability and phenotypic conformity to experimental requirements.

The plate incorporation method was used for genotoxicity assessment. 0.5 g of RFPH water extract dry powder was ground with dimethyl sulfoxide (DMSO) and diluted to 10.0 mL. After sterilization at 0.103 MPa for 20 min, the sample was serially diluted with sterile deionized water to prepare test concentrations. In the preliminary test, the TA100 strain was used without metabolic activation. No significant bacterial reverse mutation was observed at 5000 μg/plate. Based on this, formal test groups were established with doses of 62, 185, 556, 1667, and 5000 μg/plate. Control groups included negative control, solvent controls (distilled water and DMSO), and positive controls [sodium azide, dexon, 2‐acetamidofluorene, 1,8‐dihydroxyanthraquinone, and cyclophosphamide].

Three replicate plates were prepared for each strain and test concentration, with experiments conducted under both metabolic activation (S9+) and non‐metabolic activation (S9−) conditions. The verification test included dose groups of 8, 40, 200, 1000, and 5000 μg/plate, with other parameters consistent with the formal test to ensure experimental integrity. SD rat liver S9 (Cat. No. 0271041, 35 mg/mL) was purchased from IPHASE and stored at −80°C.

The number of revertant colonies for each strain (TA97a, TA98, TA100, TA102, TA1535) on histidine‐deficient medium was the primary observation index. A test substance was considered mutagenic if it caused more than a two‐fold increase in revertant colony counts compared to the blank control and showed a clear dose–response relationship, or if at least one concentration point yielded a reproducible positive response.

### 2.5 Mammalian Erythrocyte Micronucleus Test

Fifty healthy ICR mice (25–30 g, equal numbers of males and females) were randomly divided into five groups, with 10 mice per group. For the mammalian erythrocyte micronucleus test, a high‐dose group of 5 g/kg BW was used when the LD_50_ exceeded 5 g/kg BW. Three dose groups of RFPH water extract dry powder were established: low‐dose (1.25 g/kg BW), medium‐dose (2.5 g/kg BW), and high‐dose (5.0 g/kg BW), corresponding to 37.5, 75.0, and 150.0 g/kg BW of RFPH. The extract was dispersed in 1% CMC‐Na solution. The negative control group was treated with 1% CMC‐Na, and the positive control received 40 mg/kg BW cyclophosphamide. Mice were orally administered the test substance twice, 24 h apart, and euthanized 6 h after the second dose. Bone marrow was harvested from the sternum, fixed with methanol for 10 min, and stained with Giemsa for 15 min.

Under the oil immersion lens, select areas where cells are evenly distributed, staining is clear, and cell nuclei have well‐defined boundaries. After Giemsa staining, polychromatic erythrocytes (PCEs) appear grayish‐blue, while mature red blood cells (RBCs) are pink. Micronuclei are round with smooth edges, staining purple‐red or blue‐purple, and their diameter is typically 1/20 to 1/5 of the RBC diameter. According to GB 15193.5‐2014, at least 200 RBCs should be observed per animal's bone marrow sample, and the proportion of PCEs should be at least 20% of the control group. To determine the micronucleus rate (per thousand PCEs), at least 2000 PCEs per animal should be analyzed. Multiple micronuclei within a single PCE are counted as one micronucleus‐containing cell. Mean values and standard deviations are calculated by gender, and Poisson distribution is used to compare micronucleus rates between each dose group and the negative control. The test sample is considered significantly positive if any of the following conditions are met; otherwise, it is negative.

- If a dose‐dependent increase in the micronucleus cell rate is observed, and the difference compared to the control group is statistically significant, the result may be interpreted as positive.
- If the micronucleus cell rate in the experiment exhibits a statistically significant difference compared to the control group but lacks a dose–response relationship, a repeat experiment is required. If the significant difference persists in the repeated experiment, the result can be considered positive.

### 2.6 Mammalian Chromosomal Aberration Test

Twenty‐five healthy male ICR mice (25–35 g) were randomly divided into five groups, with five mice per group. For the chromosomal aberration test, a high‐dose group of 5 g/kg BW was used when the LD_50_ exceeded 5 g/kg BW. Three dose groups of RFPH water extract dry powder were established: low‐dose (1.25 g/kg BW), medium‐dose (2.5 g/kg BW), and high‐dose (5.0 g/kg BW), corresponding to 37.5, 75.0, and 150.0 g/kg BW of RFPH. The extract was dispersed in 1% CMC‐Na solution. The negative control group received 1% CMC‐Na, and the positive control received 40 mg/kg BW cyclophosphamide via a single intraperitoneal injection. Mice were orally administered the respective solution once daily for five consecutive days. The positive control group received cyclophosphamide on the first day only. Spermatocyte chromosome aberration tests were performed, and all mice were euthanized on day 12 post‐administration. Four hours before euthanasia, colchicine (6 mg/kg BW) was intraperitoneally injected. After euthanasia, testes were harvested, subjected to hypotonic treatment, and fixed with methanol: glacial acetic acid (3:1). Tissue samples were centrifuged, dropped onto slides, air‐dried, and stained with Giemsa.

Mitotic cells were examined under a 1000× magnification oil immersion microscope (Olympus, Japan). According to GB 15193.8‐2014, 100 metaphase cells per mouse were analyzed. Types and frequencies of chromosomal aberrations were documented. Statistical analysis was performed using the binomial distribution.

This study recorded the number of abnormal chromosome‐containing cells and total chromosomal aberrations in each animal. The evaluation included the types of chromosomal aberrations (fragment, minute, translocation), the proportion of abnormal cells, and the number of autosomal and sex chromosome monovalent bodies. A positive result was determined when there was a significant difference in the chromosomal aberration rate between the dose group and control, with a clear dose–response relationship.

### 2.7 Ninety‐Day Repeated‐Dose Oral Toxicity Test

#### 2.7.1 Study Design

This experiment followed the technical specifications outlined in the “National Food Safety Standard: 90‐day Repeated‐Dose Oral Toxicity Test” (GB 15193.13‐2015). Six‐week‐old SPF‐grade SD rats (70–90 g, equal numbers of males and females) were selected for the study. The experimental feed, provided by the Laboratory Animal Center of Hangzhou Medical College, complied with GB 14924.3‐2010 and contained 18% crude protein, 4% crude fat, and 5% crude fiber. Based on body weight, rats were randomly assigned to four test groups (20 rats/group, 10 males and 10 females) and two satellite groups (10 rats/group, 5 males and 5 females). The test groups received different doses of RFPH water extract dry powder (0.625, 1.25, and 2.50 g/kg BW), along with a negative control group, a satellite negative control group, and a satellite high‐dose group. The doses were incorporated into the basal diet and provided ad libitum, ensuring uniform mixing.

During the experimental period, animals were provided ad libitum access to food and water. General condition, behavior, signs of toxicity, and mortality were observed daily. The observation included coat, skin, secretions, excretions, respiratory system, nervous system, and spontaneous activity. Body weight and food intake were measured weekly, and food utilization efficiency was calculated. Ophthalmological examinations (cornea, lens, bulbar conjunctiva, iris) were performed on rats in the negative control and high‐dose groups before and at the conclusion of the experiment. If ocular changes were detected in the high‐dose group, all animals underwent ophthalmological examination. In the 7th week, urine samples were collected from satellite group rats for routine urinalysis after an overnight fast. The rats were anesthetized with 2% tribromoethyl alcohol (10 mL/kg BW), followed by blood collection from the abdominal aorta and euthanasia. Hematological and biochemical parameters were analyzed. At the end of the experiment, urine samples were collected from all groups for urinalysis after an overnight fast. The next morning, fasting body weight was measured, and rats were anesthetized with 2% tribromoethyl alcohol (10 mL/kg BW), followed by blood collection for hematological and biochemical analysis. Systemic gross pathological examinations were performed, and organ weights and organ‐to‐body weight ratios were calculated. Tissues for histopathological examination were harvested from various organs. Hematological tests were conducted using the Mindray BC5000vet analyzer, coagulation function assessed using the XL1800 coagulometer, biochemical parameters were analyzed using the LWC400 analyzer, and electrolytes were measured using the XD685 analyzer. Routine urinalysis was performed using the GF‐U180 urine analyzer, and pathological sections were stained with H&E.

#### 2.7.2 Hematological Analysis

Hematological analyses were performed using the BC5000vet automated hematology analyzer (Mindray). Parameters measured included hemoglobin concentration (HGB, g/L), white blood cell count (WBC, ×10^9^/L), red blood cell count (RBC, ×10^12^/L), lymphocyte percentage (LYM, %), granulocyte percentage (GRA, %), hematocrit (HCT, L/L), platelet count (PLT, ×10^9^/L), and intermediate cell group percentage (MID, %). The intermediate cell group included monocytes, basophils, and eosinophils. Coagulation function was assessed using the XL1800 coagulation analyzer (ZONCI Technology), with parameters including prothrombin time (PT, s) and activated partial thromboplastin time (APTT, s). Electrolyte levels (K, Na, Cl) were measured using the XD685 electrolyte analyzer (Xinda).

#### 2.7.3 Biochemical Analyzes

Blood biochemical analysis was performed using an automated biochemical analyzer (Lanyun C400). The analyzed parameters included alanine aminotransferase (ALT, U/L), alkaline phosphatase (ALP, U/L), aspartate aminotransferase (AST, U/L), gamma‐glutamyl transferase (GGT, U/L), total protein (TP, g/L), albumin (ALB, g/L), blood urea nitrogen (BUN, mmol/L), creatinine (CR, μmol/L), blood glucose (GLU, mmol/L), total cholesterol (TC, mmol/L), triglycerides (TG, mmol/L).

#### 2.7.4 Urine Routines

Urine samples were collected using metabolic cages. The experimental rats were subjected to a 12‐h fasting period (with free access to water), and individual urine samples were collected into sterile centrifuge tubes. Urine analysis was conducted using the CONTEC GF‐U180 urine analyzer, with the following parameters measured: urinary glucose (GLU), occult blood in urine (BLD), urinary protein (PRO), urine specific gravity (SG), and urine pH.

#### 2.7.5 Statistical Analysis

Statistical analysis was performed using SPSS 22.0. Levene's test was first applied to assess normality and homogeneity of variance for each variable. If normality and homogeneity were met, a one‐way ANOVA followed by Dunnett's post hoc test was used for intergroup comparisons. Otherwise, Tamhane's T2 test was applied. Data are presented as mean ± standard deviation (SD), with significance set at *p* < 0.05.

### 2.8 Teratogenicity Test

The teratogenicity test uses animal models to assess whether the dry powder of RFPH water extract induces developmental abnormalities. Pregnant animals are exposed to the test substance during the organogenesis phase, and the fetuses are examined for signs of developmental disorders or structural anomalies to evaluate the potential teratogenic effects.

SPF‐grade SD rats were used, with female rats weighing 200–240 g and male rats weighing 350–400 g. Female and male rats were co‐housed at a ratio of 2:1 overnight. Female rats with sperm detected in vaginal smears the following morning were identified as pregnant, and the day was designated as gestation day 0. The pregnant rats were randomly assigned to four groups, with at least 18 rats per group. RFPH water extract dry powder was administered at three doses (1.25, 2.5, and 5.0 g/kg BW), corresponding to 37.5, 75.0, and 150.0 g/kg BW of RFPH. The control group received distilled water. From gestation days 6 to 15, pregnant rats were orally gavaged with the test substance, while the control group received an equivalent volume of distilled water. Maternal body weight and toxicological symptoms were recorded, and body weights were measured on gestation days 0, 6, 9, 12, 15, and 20. On gestation day 20, the pregnant rats were euthanized, and uteri were excised and weighed. The number of corpora lutea, live fetuses, resorptions, and stillbirths were recorded. For each live fetus, body length, tail length, body weight, sex, and external malformations were documented. Half of the fetuses were fixed in 95% ethanol for 3 weeks and evaluated for skeletal malformations after potassium hydroxide clearing and alizarin red staining. The remaining fetuses were fixed in Bouin's solution for 2 weeks and examined for visceral malformations. Data were analyzed using SPSS 22.0, with analysis of variance and chi‐square tests applied to assess the indices.

## 3 Results

### 3.1 Acute Oral Toxicity of RFPH

The toxicological observations during the experiment revealed that no poisoning symptoms were observed in any of the tested rats or mice, and no mortality was recorded (as shown in Tables 1 and 2). Systematic gross autopsy of all experimental groups showed no morphological abnormalities in the organs. Based on the experimental data, the median lethal dose (LD_50_) of the dry powder of RFPH water extract exceeded 10.0 g/kg BW in both male and female rats and mice (equivalent to 300 g/kg BW of RFPH). According to the toxicological classification criteria, this substance is categorized as practically non‐toxic (LD_50_ > 5 g/kg BW).

**TABLE 1:** Results of the acute oral toxicity test of RFPH in mice.

Sex	Initial weight (g)	Final weight (g)	Deaths (number of deaths/group of rats)	LD_50_ (g/kg BW)
Females	20.1 ± 1.2	28.1 ± 1.3	0/10	> 10.0
Males	19.9 ± 1.3	30.9 ± 1.7	0/10	> 10.0

**TABLE 2:** Results of the acute oral toxicity test of RFPH in rats.

Sex	Initial weight (g)	Final weight (g)	Deaths (number of deaths/group of rats)	LD_50_ (g/kg BW)
Females	199.7 ± 12.4	242.9 ± 9.9	0/10	> 10.0
Males	199.1 ± 11.5	309.6 ± 17.5	0/10	> 10.0

### 3.2 Results of the Bacterial Reverse Mutation Test for RFPH

The revertant colony counts for the strains are presented in detail in Tables 3 and 4 and the experimental data indicate that, under both with and without S9 metabolic activation conditions, the revertant colony counts induced by the dry powder of RFPH water extract in each dose group did not differ significantly from those of the solvent control group. In contrast, the positive control group exhibited significant mutagenicity, with colony counts more than twice those of the solvent control group. Based on these findings, the dry powder of RFPH water extract demonstrated no genotoxic effects in the bacterial reverse mutation assay and fulfilled the negative criteria of the Ames test according to GB15193.4‐2014.

**TABLE 3:** Results of the bacterial reverse mutation test of REPH.

Substance	Dose (μg/dish)	Number of colonies (Mean ± standard deviation [SD])									
		TA_97a_		TA_98_		TA_100_		TA_102_		TA_1535_	
		−S_9_	+S_9_	−S_9_	+S_9_	−S_9_	+S_9_	−S_9_	+S_9_	−S_9_	+S_9_
Blank control	0.0	108.7 ± 13.0	148.7 ± 12.2	34.3 ± 2.5	44.7 ± 3.1	136.0 ± 12.5	178.0 ± 9.2	247.3 ± 11.5	288.0 ± 17.1	13.7 ± 2.5	16.7 ± 0.6
Distilled water	0.0	117.0 ± 14.5	149.3 ± 19.2	31.3 ± 4.2	43.0 ± 4.4	133.3 ± 12.1	170.3 ± 19.1	252.0 ± 9.2	300.7 ± 13.3	14.3 ± 1.5	15.3 ± 3.1
Dimethylsulfoxide (DMSO)	0.0	144.7 ± 19.6	133.3 ± 4.0	43.3 ± 8.3	43.3 ± 3.5	164.3 ± 23.9	161.0 ± 9.5	328.3 ± 9.3	304.0 ± 39.8	14.7 ± 1.2	17.0 ± 1.7
Dry powder of RFPH water extract	62	110.0 ± 14.4	152.7 ± 8.3	303 ± 3.1	40.3 ± 4.5	141.3 ± 17.2	166.7 ± 18.9	248.0 ± 14.0	295.3 ± 27.3	14.7 ± 1.5	15.7 ± 1.5
	185	114.0 ± 15.6	139.3 ± 9.5	31.0 ± 3.6	39.7 ± 3.1	140.7 ± 14.7	158.7 ± 12.2	245.7 ± 17.2	294.7 ± 24.2	14.7 ± 3.2	14.0 ± 2.6
	556	110.7 ± 14.0	152.7 ± 12.2	33.7 ± 3.5	41.7 ± 4.9	152.0 ± 14.4	169.3 ± 12.7	249.3 ± 15.6	282.0 ± 19.7	11.7 ± 0.6	15.0 ± 1.0
	1667	109.7 ± 10.8	143.3 ± 17.9	32.0 ± 4.4	42.0 ± 4.4	144.0 ± 14.0	175.3 ± 12.1	256.7 ± 16.7	288.7 ± 17.0	11.7 ± 0.6	14.3 ± 3.2
	5000	123.3 ± 15.5	156.7 ± 14.7	35.0 ± 4.0	43.7 ± 4.9	146.7 ± 9.0	171.3 ± 16.3	260.0 ± 15.9	304.0 ± 15.9	14.7 ± 1.5	15.3 ± 3.1
NaN_3_	1.5	/	/	/	/	2806.7 ± 150.1	/	/	/	1253.3 ± 240.1	/
Dexon	50	2446.7 ± 215.7	/	1160.0 ± 177.8	/	/	/	886.7 ± 70.2	/	/	/
2AAF	10	/	1633.3 ± 250.1	/	4933.3 ± 416.3	/	3066.7 ± 251.7	/	/	/	/
1.8HAQ	25	/	/	/	/	/	/	/	893.3 ± 61.1	/	/
CP	200	/	/	/	/	/	/	/	/	/	256.0 ± 6.9

Abbreviations: 18HAQ, 1.8‐dihydroxyanthraquinone; 2AAF, 2‐acetylaminofluorene; CP, cyclophosphamide; Dexon, sodium p‐dimethylaminobenzenediazonium sulfonate (diquat); NaN3, sodium azide.

**TABLE 4:** Results of the confirmatory bacterial reverse mutation test of REPH.

Substance	Dose (μg/dish)	Number of colonies (Mean ± standard deviation [SD])									
		TA_97a_		TA_98_		TA_100_		TA_102_		TA_1535_	
		−S_9_	+S_9_	−S_9_	+S_9_	−S_9_	+S_9_	−S_9_	+S_9_	−S_9_	+S_9_
Blank control	0.0	119.3 ± 12.1	155.0 ± 18.4	32.7 ± 4.2	41.0 ± 3.6	137.7 ± 16.6	183.3 ± 6.4	252.0 ± 14.0	304.0 ± 15.1	15.7 ± 3.2	10.7 ± 1.2
Distilled water	0.0	120.0 ± 21.6	146.0 ± 9.2	34.0 ± 3.5	43.7 ± 5.0	143.3 ± 19.0	166.0 ± 16.4	260.3 ± 15.0	287.7 ± 25.1	14.3 ± 3.2	10.7 ± 1.2
Dimethylsulfoxide (DMSO)	0.0	131.7 ± 9.7	126.3 ± 0.6	50.3 ± 2.5	37.3 ± 5.1	183.3 ± 10.0	174.0 ± 5.6	311.0 ± 27.5	264.7 ± 35.5	13.7 ± 1.2	12.7 ± 2.5
Dry powder of RFPH water extract	8	107.3 ± 14.0	140.3 ± 16.2	31.3 ± 4.2	42.0 ± 4.6	128.7 ± 14.2	169.0 ± 11.5	247.3 ± 10.1	285.3 ± 18.1	17.3 ± 1.2	13.3 ± 1.5
	40	108.7 ± 20.3	156.0 ± 15.1	33.0 ± 5.0	39.7 ± 2.5	132.3 ± 11.7	164.3 ± 17.8	254.7 ± 20.4	308.0 ± 13.1	15.7 ± 2.3	13.3 ± 0.6
	200	119.7 ± 21.5	139.0 ± 6.6	32.0 ± 2.6	42.7 ± 4.0	137.3 ± 14.0	176.7 ± 20.0	248.0 ± 14.4	294.7 ± 20.4	15.3 ± 1.5	12.0 ± 3.5
	1000	116.0 ± 11.1	152.0 ± 10.6	33.3 ± 3.2	43.3 ± 3.1	141.3 ± 12.7	170.0 ± 12.5	258.0 ± 17.1	309.3 ± 11.4	16.0 ± 2.6	12.7 ± 1.2
	5000	119.0 ± 13.0	154.7 ± 13.3	32.3 ± 4.5	42.3 ± 4.2	144.0 ± 17.4	165.7 ± 17.2	255.3 ± 10.3	302.7 ± 21.0	14.7 ± 2.1	13.0 ± 1.7
NaN_3_	1.5	/	/	/	/	2773.3 ± 117.2	/	/	/	1303.3 ± 190.4	/
Dexon	50	2620.0 ± 230.7	/	1266.7 ± 61.1	/	/	/	833.3 ± 64.3	/	/	/
2AAF	10	/	1606.7 ± 113.7	/	4866.7 ± 160.4	/	3166.7 ± 277.4	/	/	/	/
1.8HAQ	25	/	/	/	/	/	/	/	850.0 ± 79.4	/	/
CP	200	107.3 ± 14.0	140.3 ± 16.2	31.3 ± 4.2	42.0 ± 4.6	128.7 ± 14.2	169.0 ± 11.5	247.3 ± 10.1	285.3 ± 18.1	/	230.3 ± 9.9

Abbreviations: 18HAQ, 1.8‐dihydroxyanthraquinone; 2AAF, 2‐acetylaminofluorene; CP, cyclophosphamide; Dexon, sodium p‐dimethylaminobenzenediazonium sulfonate (diquat); NaN3, sodium azide.

### 3.3 Results of the Mammalian Erythrocyte Micronucleus Test for RFPH

As presented in Table 5, the data demonstrated that the PCEs/RBCs ratios in all dose groups for both genders were not less than 20% of the negative control group, indicating that the RFPH exhibited no cytotoxicity under the experimental conditions. Additionally, the micronucleus frequencies in all dose groups were not significantly different from those of the negative control group (*p* > 0.05). In contrast, the micronucleus frequency in the positive control group was significantly higher than that of the negative control group (*p* < 0.05), confirming the validity of the test system. Collectively, these findings suggest that within the tested dose range (1.25–5.0 g/kg BW, equivalent to 37.5–150 g/kg BW of RFPH), the dry powder of RFPH water extract did not induce genetic damage to mouse bone marrow erythrocytes.

**TABLE 5:** Results of the mammalian erythrocyte micronucleus test for RFPH.

Sex	Group	Dose (g/kg)	Number of animals	Number of PCEs	Number of micronucleus‐ containing PCEs	Rate of micronucle‐ ated cells (‰)	PCEs/RBCs
Females	Negative control	0.0	5	10,000	23	2.3 ± 0.6	51.7 ± 0.57
	Dry powder of RFPH water extract	1.25	5	10,000	28	2.8 ± 0.7	51.7 ± 0.57
		2.5	5	10,000	31	3.1 ± 0.4	51.9 ± 0.55
		5.0	5	10,000	30	3.0 ± 0.4	51.5 ± 0.79
	Cyclophosphamide	40 mg/kg	5	10,000	233	23.3 ± 1.2*	47.5 ± 0.79
Males	Negative control	0.0	5	10,000	27	2.7 ± 0.4	51.7 ± 0.57
	Dry powder of RFPH water extract	1.25	5	10,000	30	3.0 ± 0.8	51.2 ± 0.57
		2.5	5	10,000	27	2.7 ± 0.6	51.4 ± 0.89
		5.0	5	10,000	29	2.9 ± 0.7	51.4 ± 0.65
	Cyclophosphamide	40 mg/kg	5	10,000	230	23.0 ± 1.6*	47.6 ± 1.29

*Note:* The values are means ± standard deviation (SD), compared to the negative control group, *p < 0.05.

### 3.4 Results of Chromosomal Aberration Test in Mouse Spermatocytes for RFPH

As presented in Table 6, no statistically significant differences (*p* > 0.05) were observed in the frequency of sex chromosome monovalent bodies, autosomal monovalent bodies, chromosomal aberrations, abnormal chromosomal cells, or the rate of chromosomally abnormal cells between any of the dose groups and the negative control group following treatment with the dry powder of RFPH water extract in mouse spermatocytes. In contrast, when compared to the positive control group, all indicators were significantly higher than those of the negative control group, with a statistically significant difference (*p* < 0.01). Collectively, these findings indicate that under the experimental conditions of this study, the RFPH did not exert a significant effect on the chromosomal aberration of mouse spermatocytes.

**TABLE 6:** Results of chromosomal aberration test in mouse spermatocytes for RFPH.

Group	Dose (g/kg)	Number of cells examined	Sex chromosome monovalent body	Autosomal monovalent body	Chromosome aberration	Abnormal chromosomal cells	Rate of chromosomally abnormal cells (%)
Negative control	0.0	500	0	5	7	6	1.2
Dry powder of RFPH water extract	1.25	500	0	3	5	5	1.0
	2.5	500	0	3	6	4	0.8
	5.0	500	0	2	5	4	0.8
Cyclophosphamide	40 mg/kg	500	0	16	78	60	12.0**

*Note:* The values are means ± SD, compared to the negative control group, **p < 0.01.

### 3.5 Ninety‐Day Repeated‐Dose Oral Toxicity Study for RFPH

#### 3.5.1 Survival, Clinical Observations, and Ophthalmological Examination in a 90‐Day Repeated‐Dose Oral Toxicity Test

Throughout the 90‐day oral toxicity test period, the physiological and behavioral parameters of rats in all experimental groups, including food and water intake, fur condition, and motor coordination, remained within normal ranges. The morphological characteristics of excreta (fecal consistency and urine color) exhibited no significant variations. Behavioral monitoring revealed no abnormalities associated with neurotoxicity, such as tremors or abnormal posturing. No mortality was recorded during the entire observation period. In ophthalmological examinations, comparative analyses between the start and end of the study demonstrated that the ocular anatomical structures of rats in both the negative control group and the highest dose group remained intact. Specifically, no organic damage or functional impairments were observed in the cornea, crystalline lens, conjunctiva, iris.

#### 3.5.2 Body Weight and Body Weight Gain in a 90‐Day Repeated Oral Toxicity Test

**FIGURE 2:**
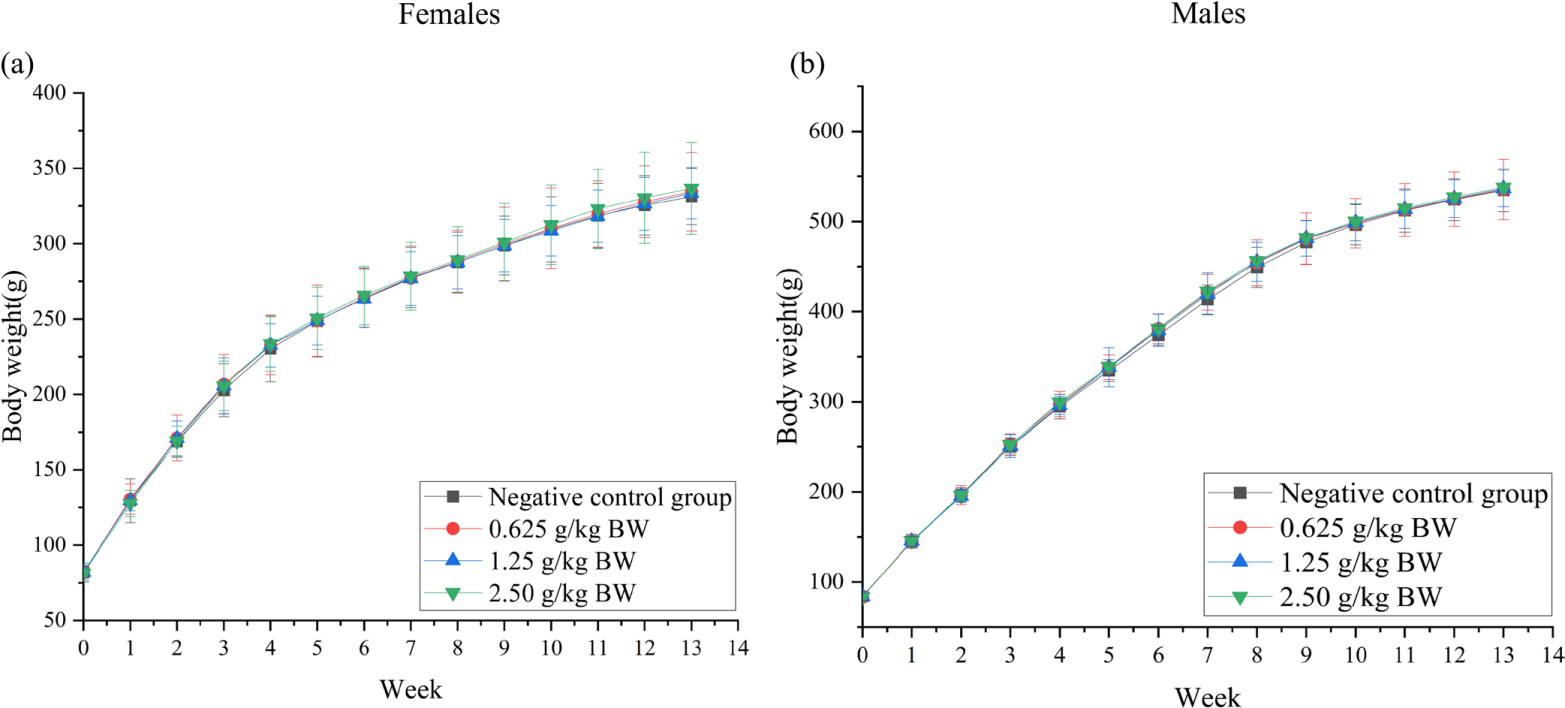
Body weight (BW) curves of female (a) and male (b) rats fed diets containing the dry powder of RFPH water extract for 90 days. Data are presented as means ± standard deviation (SD), *n* = 10 each group.

As presented in Figure 2, body weight curves of female (a) and male (b) rats fed diets containing the dry powder of RFPH water extract for 90 days. During the entire 90‐day trial period, both male and female rats in the low‐, medium‐, and high‐dose groups exhibited continuous weight gain. One‐way ANOVA analysis indicated that there were no statistically significant differences in body weight among the dose groups and negative control group (*p* > 0.05). The body weight gain trends observed in each dose group demonstrated that the incorporation of the dry powder of RFPH water extract into the diet at doses ranging from 0.625 to 2.50 g/kg BW did not exert a significant impact on the growth or metabolism of rats.

#### 3.5.3 Food Consumption and Efficiency in a 90‐Day Repeated Oral Toxicity Test

**FIGURE 3:**
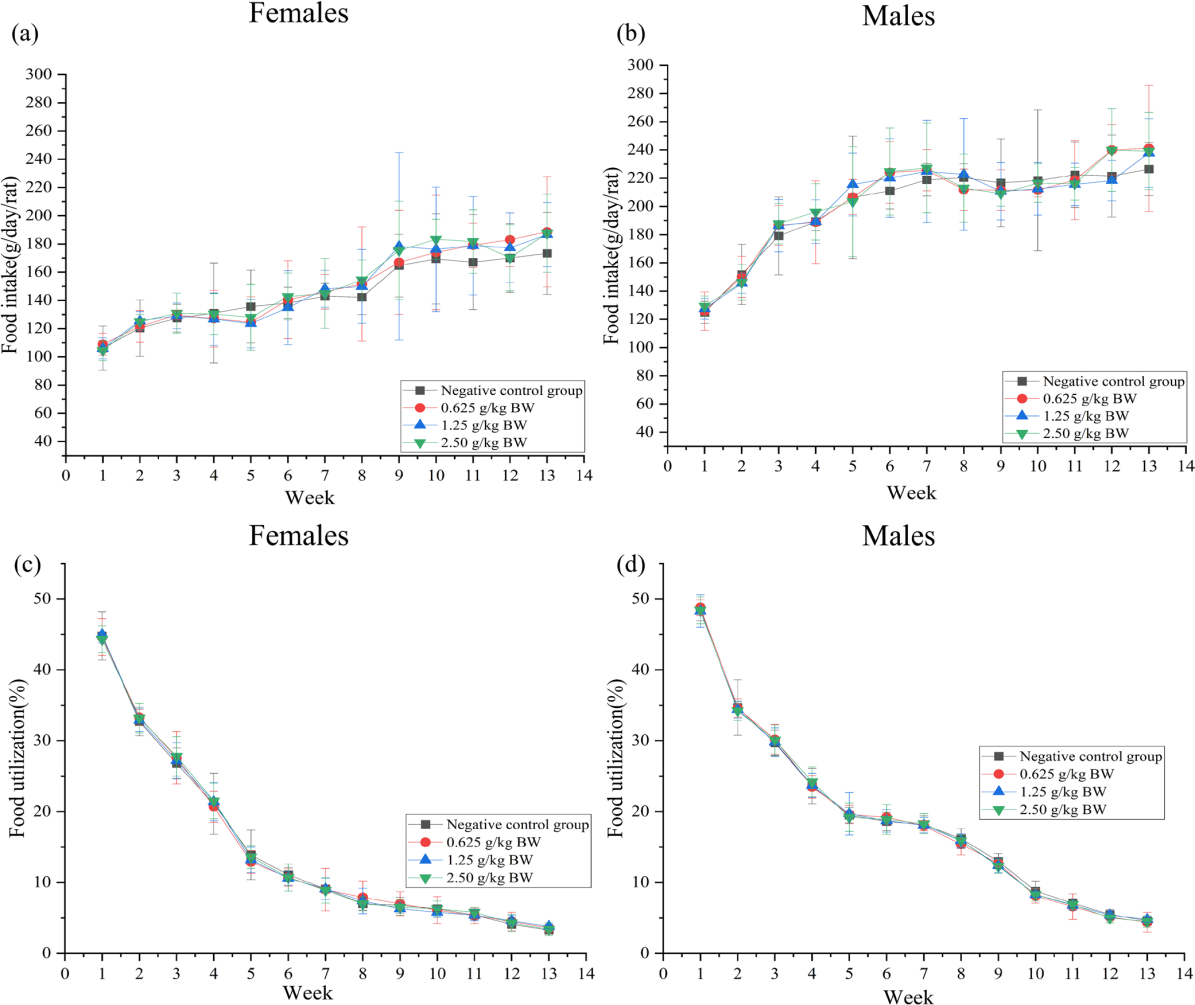
Food intake by female (a) and male (b) rats fed diets containing the dry powder of RFPH water extract for 13 weeks. Food utilization by female (c) and male (d) rats fed diets containing the dry powder of RFPH water extract for 13 weeks. Data are presented as means ± standard deviations (SDs), *n* = 10 each group. BW, body weight.

As presented in Figure 3, during the entire experimental observation period, the food intake of the test rats exhibited a progressive increase, whereas the food efficiency demonstrated a gradual decline. Statistical analysis indicated that no statistically significant differences (*p* > 0.05) were observed in food intake or food efficiency between the different dose groups of male and female rats and the negative control group.

#### 3.5.4 Hematology, Coagulation, and Biochemistry in a 90‐Day Repeated Oral Toxicity Test

As presented in Tables S1 and S2, during the medium phase of the experiment, there were no significant intergroup differences in hematological parameters and blood biochemical indicators between the high‐dose satellite group rats and the satellite control group (*p* > 0.05). As shown in Tables 7 and 8 and, at the end of the experimental period, comparisons of hematological and biochemical data between each dose experimental group and the negative control group demonstrated that all observed indicators remained statistically nonsignificant (*p* > 0.05). This study concludes that under the established experimental conditions, RFPH did not exert a significant influence on key hematological parameters, including hemoglobin concentration, red blood cell count, hematocrit, white blood cell count and differential, platelet count, prothrombin time, and activated partial thromboplastin time, nor did it affect blood biochemical indicators.

**TABLE 7:** Impact of RFPH on hematological parameters in experimental rats at the terminal stage.

	Parameter (unit)	Negative control group	0.625 g/kg BW	1.25 g/kg BW	2.50 g/kg BW
Females	HGB (g/L)	158.6 ± 7.0	154.0 ± 12.4	154.2 ± 3.7	153.4 ± 7.6
	RBC (×10^12^/L)	7.46 ± 0.37	7.20 ± 0.60	7.32 ± 0.22	7.14 ± 0.40
	WBC (×10^9^/L)	3.9 ± 1.5	4.6 ± 1.7	4.5 ± 0.9	3.5 ± 0.7
	LYM (%)	86.3 ± 4.9	82.3 ± 6.9	85.8 ± 2.9	83.0 ± 4.5
	GRA (%)	9.3 ± 4.0	13.6 ± 6.2	9.9 ± 2.2	11.6 ± 3.5
	MID (%)	4.4 ± 1.1	4.0 ± 1.4	4.4 ± 0.9	5.3 ± 1.3
	PT (s)	11.6 ± 0.7	11.6 ± 1.2	11.4 ± 0.3	11.5 ± 1.0
	APTT (s)	17.3 ± 1.3	17.2 ± 2.4	17.3 ± 1.1	17.5 ± 1.6
	PLT (×10^9^/L)	812.1 ± 126.3	877.2 ± 206.3	946.0 ± 46.9	928.2 ± 136.3
	HCT (L/L)	0.42 ± 0.02	0.41 ± 0.03	0.41 ± 0.01	0.40 ± 0.02
Males	HGB (g/L)	173.0 ± 12.3	162.9 ± 11.9	162.7 ± 8.6	164.1 ± 7.6
	RBC (×10^12^/L)	8.41 ± 0.53	8.07 ± 0.55	8.08 ± 0.41	8.18 ± 0.28
	WBC (×10^9^/L)	8.9 ± 2.0	7.3 ± 2.3	7.5 ± 2.0	7.1 ± 2.6
	LYM (%)	81.6 ± 3.1	84.7 ± 2.5	82.2 ± 4.1	82.7 ± 4.6
	GRA (%)	13.0 ± 2.5	10.4 ± 2.7	12.6 ± 3.6	12.3 ± 4.4
	MID (%)	5.5 ± 1.2	4.9 ± 0.8	5.1 ± 1.4	5.0 ± 0.8
	PT (s)	13.6 ± 1.0	13.2 ± 1.1	12.5 ± 1.0	12.7 ± 1.1
	APTT (s)	16.7 ± 1.7	15.7 ± 2.6	16.8 ± 1.8	17.3 ± 1.1
	PLT (×10^9^/L)	740.5 ± 76.8	787.3 ± 60.9	774.2 ± 71.3	765.7 ± 55.7
	HCT (L/L)	0.45 ± 0.03	0.44 ± 0.03	0.43 ± 0.02	0.43 ± 0.02

*Note:* The intermediate cell population included monocytes, eosinophils, and basophils. The values represent the mean ± standard deviation (*n* = 10), *p* > 0.05.

**TABLE 8:** Effects of RFPH on blood biochemical indices and electrolytes in experimental rats at the terminal stage.

	Parameter (unit)	Negative control group	0.625 g/kg BW	1.25 g/kg BW	2.50 g/kg BW
Females	ALT (U/L)	31.5 ± 5.3	31.7 ± 5.6	33.6 ± 5.5	36.3 ± 23.6
	AST (U/L)	95.2 ± 25.2	90.3 ± 17.7	87.7 ± 10.5	93.1 ± 25.6
	TP (g/L)	67.1 ± 3.2	67.5 ± 3.6	69.4 ± 2.9	65.5 ± 2.2
	Alb (g/L)	31.2 ± 2.0	30.5 ± 2.0	32.5 ± 2.0	30.9 ± 1.6
	TC (mmol/L)	1.67 ± 0.32	1.88 ± 0.47	1.80 ± 0.24	1.83 ± 0.31
	TG (mmol/L)	0.17 ± 0.02	0.22 ± 0.08	0.21 ± 0.04	0.18 ± 0.04
	Glu (mmol/L)	5.56 ± 0.76	5.23 ± 0.87	5.24 ± 0.83	5.63 ± 0.72
	BUN (mmol/L)	6.02 ± 1.26	6.51 ± 1.27	7.16 ± 2.37	5.92 ± 0.79
	CR (μmol/L)	50.1 ± 7.2	51.9 ± 5.9	51.6 ± 3.1	49.3 ± 2.9
	GGT (U/L)	10.6 ± 0.6	10.7 ± 1.4	10.0 ± 1.0	10.0 ± 0.7
	ALP (U/L)	44.7 ± 11.3	55.0 ± 18.1	56.7 ± 17.8	50.4 ± 15.8
	K (mmol/L)	4.09 ± 0.34	3.92 ± 0.24	3.95 ± 0.27	4.20 ± 0.95
	Na (mmol/L)	146.2 ± 2.4	145.3 ± 1.7	146.3 ± 0.9	145.9 ± 0.9
	Cl (mmol/L)	110.3 ± 2.2	109.2 ± 2.2	109.9 ± 1.8	109.5 ± 1.5
Males	ALT (U/L)	42.7 ± 6.1	40.3 ± 7.0	40.6 ± 6.3	40.9 ± 7.5
	AST (U/L)	118.6 ± 21.0	110.5 ± 26.6	109.8 ± 16.3	103.6 ± 13.1
	TP (g/L)	60.2 ± 2.2	60.8 ± 2.0	61.2 ± 2.5	62.4 ± 4.3
	Alb (g/L)	25.6 ± 0.9	25.3 ± 1.1	25.2 ± 1.0	25.0 ± 1.8
	TC (mmol/L)	1.38 ± 0.16	1.69 ± 0.28	1.59 ± 0.41	1.58 ± 0.42
	TG (mmol/L)	0.32 ± 0.12	0.39 ± 0.19	0.40 ± 0.22	0.33 ± 0.19
	Glu (mmol/L)	5.27 ± 0.84	5.68 ± 0.65	5.90 ± 0.68	5.66 ± 0.93
	BUN (mmol/L)	4.95 ± 0.86	4.81 ± 0.69	4.22 ± 0.51	4.95 ± 0.75
	CR (μmol/L)	46.3 ± 2.4	45.1 ± 2.6	44.9 ± 3.0	45.7 ± 3.7
	GGT (U/L)	9.0 ± 1.4	9.2 ± 1.1	8.6 ± 0.7	8.4 ± 0.8
	ALP (U/L)	89.4 ± 11.7	92.5 ± 15.9	83.6 ± 18.3	89.8 ± 16.8
	K (mmol/L)	4.99 ± 0.49	4.95 ± 0.32	4.87 ± 0.24	5.13 ± 0.66
	Na (mmol/L)	146.7 ± 1.3	145.7 ± 1.2	146.7 ± 1.4	146.6 ± 1.0
	Cl (mmol/L)	109.6 ± 1.9	109.1 ± 1.3	108.2 ± 1.3	109.2 ± 1.3

*Note:* The values are mean ± SD, *n* = 10, compared to negative control group, *P* > 0.05.

#### 3.5.5 Organ Weights in a 90‐Day Repeated Oral Toxicity Test

On day 90 of the experiment, the final body weight and the weights of the brain, heart, liver, kidneys, spleen, uterus, ovaries, thymus, and adrenal glands in each dose group remained comparable to those of the negative control group, with no statistically significant differences detected. As presented in Tables S3 and S4, no statistically significant differences were observed in the organ weights or organ‐to‐body weight ratios of male and female rats across all dose groups compared to the negative control group (*p* > 0.05).

#### 3.5.6 Urinalysis in a 90‐Day Repeated Oral Toxicity Test

As indicated by the Table S5, during the mid‐phase of the experiment, no statistically significant differences were observed in urine test parameters between the high‐dose satellite group and the negative control group (*p* > 0.05). At the end of the experiment, comparisons of urine test parameters between each dose group and the negative control group also revealed no significant differences (Tables 9 and 10, *p* > 0.05). These findings suggested that the intervention with RFPH did not exert a measurable influence on the routine urine test results of the experimental rats under the established experimental conditions.

**TABLE 9:** Effects of RFPH on urinalysis parameters in female rats at terminal stage (*n* = 10).

	Parameter	Degree	Negative control group	0.625 g/kg BW	1.25 g/kg BW	2.50 g/kg BW
Females	Glu	−	10	10	10	10
		±	0	0	0	0
	PRO	−	3	3	1	0
		±	6	7	8	9
		1+	1	0	1	1
	BLD	−	10	10	10	10
		±	0	0	0	0
	SG	1.005	6	4	4	7
		1.010	3	5	3	2
		1.015	1	1	3	1
		1.020	0	0	0	0
	pH	7.0	2	1	0	2
		7.5	1	2	4	4
		8.0	6	5	5	4
		8.5	1	2	1	0

Abbreviations: BLD, urine occult blood; Glu, urine glucose; pH, urine pH; PRO, urine protein; SG, urine specific gravity.

**TABLE 10:** Effects of RFPH on urinalysis parameters in male rats at terminal stage (*n* = 10).

	Parameter	Degree	Negative control group	0.625 g/kg BW	1.25 g/kg BW	2.50 g/kg BW
Males	Glu	−	10	10	10	10
		±	0	0	0	0
	PRO	−	0	1	1	0
		±	6	4	6	8
		1+	4	5	3	2
	BLD	−	8	9	9	8
		±	2	0	0	2
		1+	0	1	1	0
	SG	1.005	7	4	7	4
		1.010	3	4	3	3
		1.015	0	2	0	3
		1.020	0	0	0	0
	pH	6.5	0	0	0	0
		7.0	1	1	0	1
		7.5	3	1	4	0
		8.0	3	4	6	5
		8.5	3	4	0	4

Abbreviations: BLD, urine occult blood; Glu, urine glucose; pH, urine pH; PRO, urine protein; SG, urine specific gravity.

#### 3.5.7 Histopathology in a 90‐Day Repeated Oral Toxicity Test for RFPH

In the 90‐day oral toxicity study, gross anatomical and histopathological examinations were performed on 80 rats (equally divided between males and females) from the negative control group and three dose groups. Gross anatomical observations revealed no apparent abnormalities in the color, size, or morphological structure of the major organs across all groups. The histopathological examination of 20 rats (equally divided between males and females) from the high‐dose group and the negative control group demonstrated that the tissues including the heart, stomach, duodenum, jejunum, ileum, colon, rectum, pancreas, bladder, brain, thyroid, adrenals, hypophysis, spleen, lymph nodes, ovary, uterus, testes, and epididymides were normal. Histopathological examination revealed the following findings: A small amount of inflammatory cell infiltrations around the hepatic lobular vessels were observed in 1 male and 1 female in the control group, as well as in 1 female in the highest dose group; A small amount of diffuse or focal inflammatory cell infiltrations around the bronchi in lungs were observed in 2 males and 1 female in the control group, as well as 1 male and 1 female in the highest dose group; A small amount of inflammatory cell infiltrations in the renal was observed in 1 female in the control group, as well as a small amount of basophilic change and vacuolation in epithelial cells of renal tubules were observed in 1 male in the highest dose group (Table 11); A few animals exhibited mild inflammatory cell infiltration and other spontaneous lesions; however, no characteristic pathological changes attributable to the test substance were observed. Based on the historical control data obtained from our laboratory, all observed findings were consistent with the typical background lesions present in normal rats of the same age and strain used in this study. These findings were considered to be spontaneous and/or incidental in nature and were not considered to be related to the administered treatment.

**TABLE 11:** Impact of RFPH on histopathology in experimental male rats at the terminal stage.

Organs	Observations	Male		Female	
		Control	2.5 g/kg BW	Control	2.5 g/kg BW
Liver	Inflammatory cell infiltrations around the hepatic lobular vessels	1/10	0/10	1/10	1/10
Lungs	Diffuse or focal inflammatory cell infiltrations around the bronchi in lungs	2/10	1/10	1/10	1/10
Kidney	Inflammatory cell infiltrations in the renal	0/10	0/10	1/10	0/10
	Basophilic change and vacuolation in epithelial cells of renal tubules	0/10	1/10	0/10	0/10

### 3.6 Teratogenicity Test of RFPH

In this experiment, at least 18 fertilized female rats were initially allocated to each group. After excluding the nonpregnant fertilized rats, at least 16 pregnant rats remained in each group, and all experimental indicators from these pregnant rats were included in the statistical analysis. On days 0, 6, 9, 12, 15, and 20 postfertilization, the body weight, weight gain, and net weight gain of pregnant rats in each dose group exhibited no statistically significant differences compared with the control group (Table S6, *p* > 0.05). With the key indicators such as the number of corpus luteum, the number of implantations, the number of resorptions, the number of live fetuses, and the number of dead fetuses, each dose group was comparable to that of the control group (Table 12), with no statistically significant differences observed (*p* > 0.05). These findings suggest that under the experimental conditions employed in this study, administration of RFPH did not result in any significant impairment of reproductive function in pregnant rats.

**TABLE 12:** Effects on reproductive function in pregnant rats (mean ± SD).

Group	Number of fertilized rats	Pregnant rats (%)	Number of corpora lutea	Implantation (%)	Resorption (%)	Dead fetuses (%)	Live fetuses (%)	Average live fetuses per litter (mean ± SD)
Control group	19	17 (89.5)	318	260 (81.8)	10 (3.8)	0 (0.0)	250 (96.2)	14.7 ± 2.3
1.25 g/kg BW	18	16 (88.9)	297	245 (82.5)	7 (2.9)	1 (0.4)	237 (96.7)	14.8 ± 2.5
2.5 g/kg BW	19	19 (100.0)	367	309 (84.2)	13 (4.2)	0 (0.0)	296 (95.8)	15.6 ± 2.3
5.0 g/kg BW	19	17 (89.5)	302	251 (83.1)	8 (3.2)	0 (0.0)	243 (96.8)	14.3 ± 3.7

Regarding fetal growth and development (Table 13), growth parameters such as total body length (body length + tail length), body weight, and sex ratio of fetal rats in each dose group showed no statistically significant differences compared with the control group (*p* > 0.05). Collectively, these results indicated that under the experimental conditions of this study, administration of RFPH had no discernible effect on the growth and development of fetal mice. As presented in Tables 14, 15, 16, no external or internal malformations were observed in the fetuses of any dose group or the control group (*p* > 0.05).

**TABLE 13:** Effects on growth and development in fetal rats (mean ± SD).

Group	Number of examined fetal rats	Body length (cm)	Body weight (g)	Female:Male
Control group	250	5.233 ± 0.156	3.80 ± 0.23	1:1
1.25 g/kg BW	237	5.270 ± 0.149	3.85 ± 0.21	1:1.13
2.5 g/kg BW	296	5.239 ± 0.272	3.91 ± 0.72	1:1.13
5.0 g/kg BW	243	5.201 ± 0.151	3.78 ± 0.22	1:0.84

**TABLE 14:** Effects on external malformations in fetal rats.

Group	Number of examined fetal rats	External malformation rate (%)				
		Exencephaly	Spina bifida	Gastroschisis	Cleft lip	No tail
Control group	250	0 (0.0)	0 (0.0)	0 (0.0)	0 (0.0)	0 (0.0)
1.25 g/kg BW	237	0 (0.0)	0 (0.0)	0 (0.0)	0 (0.0)	0 (0.0)
2.5 g/kg BW	296	0 (0.0)	0 (0.0)	0 (0.0)	0 (0.0)	0 (0.0)
5.0 g/kg BW	243	0 (0.0)	0 (0.0)	0 (0.0)	0 (0.0)	0 (0.0)

**TABLE 15:** Effects on skeletal malformations in fetal rats.

Group	Number of examined fetal rats	Skeletal malformation rate (%)					
		Rib malformations	Posterior parietal bone ossification incomplete	Cervical vertebra absence	Sacral vertebra absence	Occipital bone absence	Fontanelle enlargement
Control group	129	0 (0.0)	0 (0.0)	0 (0.0)	0 (0.0)	0 (0.0)	0 (0.0)
1.25 g/kg BW	123	0 (0.0)	0 (0.0)	0 (0.0)	0 (0.0)	0 (0.0)	0 (0.0)
2.5 g/kg BW	154	0 (0.0)	0 (0.0)	0 (0.0)	0 (0.0)	0 (0.0)	0 (0.0)
5.0 g/kg BW	127	0 (0.0)	0 (0.0)	0 (0.0)	0 (0.0)	0 (0.0)	0 (0.0)

**TABLE 16:** Effects on visceral malformations in fetal rats.

Group	Number of examined fetal rats	Visceral malformation rate (%)			
		Nasal congestion	Ventricular congestion	Kidney atrophy	Cleft palate
Control group	121	0 (0.0)	0 (0.0)	0 (0.0)	0 (0.0)
1.25 g/kg BW	114	0 (0.0)	0 (0.0)	0 (0.0)	0 (0.0)
2.5 g/kg BW	142	0 (0.0)	0 (0.0)	0 (0.0)	0 (0.0)
5.0 g/kg BW	116	0 (0.0)	0 (0.0)	0 (0.0)	0 (0.0)

## 4 Discussion

The safety assessment of new food ingredients is important for expanding sustainable food resources and addressing global nutritional demands. New food ingredients refer to those that have no established history of consumption in China. These may include animals, plants, microorganisms and their derived components, as well as food substances produced using novel processing techniques. However, RFPH has not been classified as a new food ingredient, which significantly restricts industrial development. This study comprehensively evaluated the toxicological profile of RFPH in accordance with the Chinese national food safety standard for the safety assessment of new food ingredients. Our findings demonstrate no evidence of significant toxicity in acute oral, genotoxicity (Ames test, micronucleus, and chromosomal aberration assays), subchronic 90‐day oral, or teratogenicity studies. The absence of adverse effects, mortality, genotoxic potential, target organ toxicity, or developmental abnormalities across these assessments supports the conclusion that RFPH poses negligible toxicological risk under the tested conditions. These collective results provide information for its potential development and regulatory consideration as a novel food ingredient.

*Ficus pandurata* Hance var. *angustifolia* Cheng is morphologically similar to *Ficus pandurata* Hance and *Ficus pandurata* Hance var. *holophylla Migo*, which belong to the Moraceae family and the genus *Ficus* (Zhejiang Medicinal Plants Editorial Group 1980). Their primary distinctions are leaf morphology, whereas other morphological characteristics are largely consistent. However, potential differences in their chemical composition and medicinal efficacy have yet to be fully elucidated (Lu and Peng 2018). The edible and medicinal uses of the RFPH have primarily been transmitted orally across generations in China, with limited documentation available. Due to an insufficient understanding of its toxicological profile, the widespread application of RFPH in the food industry remains limited. According to Chinese consumption records, 25 g of RFPH is typically used for every 1 kg of goose meat, corresponding to an estimated daily edible intake of 5–15 g per person. According to the quality standard for RFPH specified in the 2015 edition of the “Zhejiang Province Chinese Medicinal Materials Processing Specifications,” the recommended dosage ranges of RFPH from 10 to 30 g (Zhejiang Food and Drug Administration 2016). In our acute toxicity tests, LD_50_ of the dry powder of RFPH water extract (equivalent to 300 g/kg BW of RFPH) exceeded 10.0 g/kg BW in both male and female rats and mice. According to the toxicological classification criteria, this RFPH is categorized as practically non‐toxic.

Previous studies have demonstrated that RFPH is rich in proteins, crude fats, crude fibers, and a variety of essential trace elements (Ying et al. 2012; Zhang et al. 2015). RFPH contains abundant Cr (chromium), Mn (manganese), Fe (iron), Cu (copper), Zn (zinc), Se (selenium), Ag (silver), and Cd (cadmium), with relatively higher concentrations of Mn, Fe, and Zn. The levels of Pb (lead), As (arsenic), and Hg (mercury) remain within the regulatory limits set by Chinese standards (Zhang et al. 2015). However, neither domestic nor international published studies, including our own research, have detected aluminum content in RFPH. The JECFA has indicated that the recommended weekly intake limit of Al (aluminum) for humans is 2 mg/kg BW (Jiang et al. 2023). Excessive Al intake may lead to damage to the liver, kidneys, and spleen. In our study, no evidence of liver, kidney, or spleen damage was found to be associated with RFPH exposure. However, the Al content in RFPH needs to be further analyzed.

Current evidence indicates that *Ficus pandurata* Hance var. *angustifolia* Cheng predominantly comprises a variety of bioactive chemical constituents, including flavonoids, phenolic compounds, and terpenoids (Zhang et al. 2015). Phytochemical investigations on the RFPH have primarily concentrated on flavonoids and polysaccharides. In contrast, studies on its congeneric species *Ficus pandurata* Hance have led to the isolation and identification of alkaloidal constituents (Khedr et al. 2018). Based on taxonomic characteristics, phylogenetic relationships, and the biosynthetic potential of secondary metabolites within the genus, the RFPH is postulated to possess theoretical capacity for alkaloid biosynthesis. While alkaloids exhibit significant pharmacological activities, they may also pose potential toxicological risks, such as central nervous system depression, respiratory suppression, and hepatorenal toxicity (Ling et al. 2022; Gardiner et al. 2016; Hamed et al. 2021).

*Ficus pandurata* Hance var. *angustifolia* Cheng is predominantly found in natural habitats such as hillsides, roadsides, and open fields (Ying et al. 2012). Although significant progress has been made in the development of an in vitro rapid propagation system for *Ficus pandurata* Hance var. *angustifolia* Cheng (Liu et al. 2010), the current utilization of this species remains heavily reliant on wild resources. Artificial cultivation has yet to overcome the technical challenges associated with large‐scale production, and a comprehensive food industrial application framework remains to be established.

In conclusion, in the acute oral toxicity test, no significant poisoning symptoms or deaths were observed in either the tested rats or mice. Furthermore, no abnormal pathological changes were detected in the organs of animals from any experimental group upon dissection. The LD_50_ values of the dry powder of RFPH water extract exceeded 10.0 g/kg BW in all cases. According to the toxicity classification standard, this substance was categorized as practically non‐toxic. The results of the bacterial reverse mutation test, mammalian micronucleus test, and mouse chromosomal aberration test were all negative, indicating that *Ficus pandurata* Hance var. *angustifolia* Cheng exhibited no mutagenic effects under the experimental conditions employed. In the 90‐day oral toxicity test, SD rats were administered doses of 0.625, 1.25, and 2.50 g/kg BW/day. No toxic signs or symptoms were observed during the study period.

While this study provides valuable information regarding the toxicological profile of RFPH, several limitations must be considered. The experiments focused on the water extract, which may not fully represent the complete chemical profile of RFPH, as other extracts (e.g., alcohol or ether extracts) may exhibit different biological activities. Water extracts were used because they reflect real‐world soup preparation and local traditional use, and their solubility during cooking better represents actual toxicity and bioactivity. Additionally, the current study does not evaluate the long‐term, low‐dose exposure or assess potential cumulative effects of RFPH in human consumption scenarios, which should be further explored in future research. Furthermore, while the study confirms the absence of acute toxicity, the potential effects of chronic exposure remain unexamined.

Future studies should extend the investigation to other extracts of RFPH, comparing their biological activities and toxicological profiles. Additionally, more research is needed to explore the long‐term effects of RFPH water extract, especially regarding cumulative exposure, as well as its effects on specific organs and systems. Given the potential for alkaloid biosynthesis in RFPH, it is crucial to investigate the presence and effects of alkaloids in the plant. Finally, clinical studies and human trials are needed to verify the safety of RFPH consumption across different populations, including sensitive groups such as pregnant women and children, to ensure its safe and sustainable application as a novel food ingredient.

## Author Contributions

**Yamin Chen:** data curation. **Siyuan Wang:** validation. **Ye Jin:** funding acquisition. **Jianguo Chen:** validation. **Junjie Pan:** investigation. **Fangfang Ma:** project administration. **Kejun Cheng:** funding acquisition. **Xuefeng Qu:** conceptualization, writing – review and editing. **Zhijing Fu:** writing – original draft. **Yangjunna Zhang:** validation. **Huaxing Luo:** methodology, validation. **Zhen Liu:** methodology, did experiment. **Jingjin Hu:** did experiment.

## Funding

This work was supported in part by the Key Subjects of Nutrition of Zhejiang Province [16‐zc03], as well as by Lishui University and Agriculture and Rural Bureau of Suichang County for “Basic Research Project for New Resource Food Application of *Ficus pandurata* Hance var. *angustifolia* Cheng (Xiaoxianggou)” [HZCB‐2021009].

## Ethics Statement

In accordance with the “Guidelines for Ethical Review of Animal Welfare in Experimental Animals” (GB/T 35892‐2018, revised in 2018), the experimental protocol was submitted to and approved by the Animal Care and Use Committee of Hangzhou Medical College (approval number: ZJCLA‐IACUC20100008). All animal procedures were conducted in compliance with national laws and regulations, ensuring that animal welfare was prioritized and that any potential suffering was minimized throughout the experiment.

## Conflicts of Interest

The authors declare no conflicts of interest.

## Supporting information


**Table S1:** Impact of RFPH on hematological parameters in experimental rats at the medium stage (mean ± SD, *n* = 5).
**Table S2:** Effects on blood biochemical indices and electrolyte in experimental rats at the medium stage for RFPH (mean ± SD, *n* = 5).
**Table S3:** Effects on organ weights in experimental rats for RFPH at the terminal stage (g, mean ± s, *n* = 10).
**Table S4:** Effects on organ‐to‐body weight ratio in experimental rats for RFPH at the terminal stage (%, mean ± s, *n* = 10).
**Table S5:** Effects of RFPH on urine test results in experimental rats at the medium stage (*n* = 5).
**Table S6:** Effects on body weight in pregnant rats.

## Data Availability

The datasets used and/or analyzed during the current study are available from the corresponding author upon reasonable request.

## References

[fsn371979-bib-0001] Alkhatib, A. , C. Tsang , A. Tiss , et al. 2017. “Functional Foods and Lifestyle Approaches for Diabetes Prevention and Management.” Nutrients 9, no. 12: 1310. 10.3390/nu9121310. 29194424 PMC5748760

[fsn371979-bib-0002] Chen, C. , X. J. Hu , L. Y. Song , W. B. Dai , R. M. Yu , and W. W. Peng . 2020. “Research Progress on *Ficus pandurata* Hance.” Food and Drug 22, no. 2: 148–153.

[fsn371979-bib-0003] Cheng, K. J. , Q. D. Lv , X. W. Li , and W. L. Cheng . 2025. Standardized Cultivation of Characteristic Chinese Medicinal Materials in Eastern China Mountainous Areas. Science China Press.

[fsn371979-bib-0004] Dias, M. C. , D. C. G. A. Pinto , and A. M. S. Silva . 2021. “Plant Flavonoids: Chemical Characteristics and Biological Activity.” Molecules (Basel, Switzerland) 26, no. 17: 5377. 10.3390/molecules26175377. 34500810 PMC8434187

[fsn371979-bib-0005] Editorial Committee of Flora Republicae Popularis Sinicae, Chinese Academy of Sciences . 2004. Flora Republicae Popularis Sinicae. Vol. 23, 154. Science China Press.

[fsn371979-bib-0006] Gardiner, S. J. , A. B. Chang , J. M. Marchant , and H. L. Petsky . 2016. “Codeine Versus Placebo for Chronic Cough in Children.” Cochrane Database of Systematic Reviews 7, no. 7: CD011914. 10.1002/14651858.CD011914.pub2. 27405706 PMC6457872

[fsn371979-bib-0007] Ghorbani, A. 2017. “Mechanisms of Antidiabetic Effects of Flavonoid Rutin.” Biomedicine & Pharmacotherapy 96: 305–312. 10.1016/j.biopha.2017.10.001. 29017142

[fsn371979-bib-0008] Hamed, M. A. , G. O. Aremu , and R. E. Akhigbe . 2021. “Concomitant Administration of HAART Aggravates Anti‐Koch‐Induced Oxidative Hepatorenal Damage via Dysregulation of Glutathione and Elevation of Uric Acid Production.” Biomedicine & Pharmacotherapy 137: 111309. 10.1016/j.biopha.2021.111309. 33524784

[fsn371979-bib-0009] Jiang, H. , L. M. Huang , L. L. Wang , H. Liu , and B. Zhu . 2023. “Risk Assessment of Dietary Aluminum Exposure Among Residents in Hangzhou City.” Chinese Journal of Food Hygiene 35, no. 2: 224–229. 10.13590/j.cjfh.2023.02.012.

[fsn371979-bib-0010] Khedr, A. I. M. , S. R. M. Ibrahim , G. A. Mohamed , S. A. Ross , and K. Yamada . 2018. “Panduramides A‐D, New Ceramides From *Ficus pandurata* Fruits.” Phytochemistry Letters 23: 100–105.

[fsn371979-bib-0011] Ling, S. , F. Ceban , L. M. W. Lui , et al. 2022. “Molecular Mechanisms of Psilocybin and Implications for the Treatment of Depression.” CNS Drugs 36, no. 1: 17–30. 10.1007/s40263-021-00877-y. 34791625

[fsn371979-bib-0012] Liu, C. H. , X. Lun , and G. H. Xia . 2010. “Tissue Culture and Rapid Propagation of *Ficus pandurata* Hance var. *angustifolia* Cheng.” Plant Physiology Communications 46, no. 6: 603–604. 10.13592/j.cnki.ppj.

[fsn371979-bib-0013] Liu, F. , X. Zhang , B. Zhao , X. Tan , L. Wang , and X. Liu . 2019. “Role of Food Phytochemicals in the Modulation of Circadian Clocks.” Journal of Agricultural and Food Chemistry 67, no. 32: 8735–8739. 10.1021/acs.jafc.9b02263. 31244204

[fsn371979-bib-0014] Lu, Y. C. , and W. W. Peng . 2018. “Textual Research on Varieties and Modern Research Progress of *Ficus pandurata* Hance.” Asia‐Pacific Traditional Medicine 14, no. 10: 84–87.

[fsn371979-bib-0016] Serafini, M. , I. Peluso , and A. Raguzzini . 2010. “Flavonoids as Anti‐Inflammatory Agents.” Proceedings of the Nutrition Society 69, no. 3: 273–278. 10.1017/S002966511000162X. 20569521

[fsn371979-bib-0017] Walum, E. 1998. “Acute Oral Toxicity.” Environmental Health Perspectives 106, no. Suppl 2: 497–503. 10.1289/ehp.98106497. 9599698 PMC1533392

[fsn371979-bib-0018] Ying, Y. Y. , X. Z. Wang , and G. Q. He . 2012. “Analysis of Nutritional Components and Determination of Flavonoid Content in *Ficus pandurata* Hance Var. *angustifolia* Cheng.” Science and Technology of Food Industry 33, no. 14: 90–92+99. 10.13386/j.issn1002-0306.2012.14.010.

[fsn371979-bib-0019] Zeiger, E. 1998. “Identification of Rodent Carcinogens and Noncarcinogens Using Genetic Toxicity Tests: Premises, Promises, and Performance.” Regulatory Toxicology and Pharmacology: RTP 28, no. 2: 85–95. 10.1006/rtph.1998.1234. 9927558

[fsn371979-bib-0020] Zhang, X. P. , K. Z. Jiang , H. Q. Lv , J. Nie , and Z. G. Li . 2015. “Identification of Major Chemical Constituents in the Ethyl Acetate Extract of *Ficus pandurate* Hance Var. *angustifolia* Cheng Roots and Stems by HPLC‐Q‐TOF MS.” Chinese Journal of Mass Spectrometry 36, no. 4: 310–320.

[fsn371979-bib-0021] Zhang, Y. , J. Pan , Y. Liu , X. Zhang , and K. Cheng . 2023. “Effects of *Ficus pandurata* Hance var. *angustifolia* Cheng Flavonoids on Intestinal Barrier and Cognitive Function by Regulating Intestinal Microbiota.” Foods (Basel, Switzerland) 12, no. 8: 1682. 10.3390/foods12081682. 37107477 PMC10137925

[fsn371979-bib-0022] Zhejiang Food and Drug Administration . 2016. Zhejiang Provincial Standards of Processing Chinese Crude Drugs 2015 Edition. China Medical Science Press.

[fsn371979-bib-0023] Zhejiang Medicinal Plants Editorial Group . 1980. Zhejiang Medicinal Plants. Zhejiang Science and Technology.

