# Supplementary material for: Toxicological Evaluation of the Roots of Ficus pandurata Hance var. angustifolia Cheng
Source: Food Sci Nutr. 2026 Jun 2;14(6):e71979. doi: 10.1002/fsn3.71979 (PMC13240308; doi:10.1002/fsn3.71979)
Supplement: Supplementary file 1 — Table S1: Impact of RFPH on hematological parameters in experimental rats at the medium stage (mean ± SD, n = 5). Table S2: Effects on blood biochemical indices and electrolyte in experimental rats at the medium stage for RFPH (mean ± SD, n = 5). Table S3: Effects on organ weights in experimental rats for RFPH at the terminal stage (g, mean ± s, n = 10). Table S4: Effects on organ‐to‐body weight ratio in experimental rats for RFPH at the terminal stage (%, mean ± s, n = 10). Table S5: Effects of RFPH on urine test results in experimental rats at the medium stage (n = 5). Table S6: Effects on body weight in pregnant rats. [file FSN3-14-e71979-s001.docx]

Suppl. Table 1 Impact of RFPH on hematological parameters in experimental rats at the medium stage (mean ± SD, n = 5)

| Parameter  (unit) | Females | | Males | |
| --- | --- | --- | --- | --- |
|  | Satellite control group | Satellite high-dose group | Satellite control group | Satellite high-dose group |
| HGB (g/L) | 119.0±17.0 | 117.4±15.2 | 111.6±6.6 | 129.8±16.5 |
| RBC(×10^12^/L) | 5.36±0.87 | 5.28±0.75 | 5.15±0.31 | 6.02±0.88 |
| WBC(×10^9^/L) | 4.7±1.3 | 3.9±0.8 | 5.5±1.6 | 5.6±1.8 |
| LYM(%) | 87.7±5.0 | 85.4±2.5 | 87.3±1.4 | 89.6±2.0 |
| GRA(%) | 8.3±3.6 | 10.8±1.9 | 8.2±1.2 | 6.7±1.3 |
| MID(%) | 4.1±1.7 | 3.8±0.8 | 4.4±0.7 | 3.7±0.9 |
| PT (s) | 13.4±1.0 | 14.3±1.7 | 14.2±1.3 | 13.8±0.7 |
| APTT (s) | 19.7±2.5 | 20.1±1.9 | 20.0±1.2 | 19.2±2.3 |
| PLT(×10^9^/L) | 672.2±105.6 | 776.6±182.5 | 656.6±108.7 | 656.8±50.3 |
| HCT (L/L) | 0.33±0.05 | 0.33±0.04 | 0.31±0.02 | 0.35±0.04 |

Suppl. Table 2 Effects on blood biochemical indices and electrolyte in experimental rats at the medium stage for RFPH (mean ± SD, n = 5).

| Parameter  (unit) | Females | | Males | |
| --- | --- | --- | --- | --- |
|  | Satellite control group | Satellite high-dose group | Satellite control group | Satellite high-dose group |
| ALT (U/L) | 37.2±7.1 | 37.9±6.6 | 42.3±4.3 | 43.9±8.2 |
| AST (U/L) | 109.2±18.5 | 114.0±16.0 | 110.8±26.7 | 124.2±23.8 |
| TP (g/L) | 61.8±2.6 | 64.2±1.0 | 59.1±2.2 | 59.4±3.0 |
| Alb (g/L) | 26.3±1.0 | 26.7±1.2 | 23.9±1.2 | 24.3±1.0 |
| TC (mmol/L) | 1.73±0.23 | 1.84±0.44 | 1.52±0.10 | 1.62±0.13 |
| TG (mmol/L) | 0.26±0.07 | 0.35±0.27 | 0.46±0.35 | 0.55±0.19 |
| Glu (mmol/L) | 5.60±0.78 | 6.17±0.92 | 5.95±0.50 | 6.11±0.26 |
| BUN (mmol/L) | 6.86±2.45 | 5.00±0.32 | 5.57±1.03 | 6.00±0.67 |
| CR (μmol/L) | 46.6±7.3 | 45.3±3.3 | 43.8±2.4 | 45.2±2.8 |
| GGT (U/L) | 9.7±0.8 | 9.2±0.6 | 9.0±1.4 | 9.6±0.6 |
| ALP (U/L) | 119.5±28.0 | 121.5±31.2 | 163.4±24.1 | 153.1±29.8 |
| K (mmol/L) | 4.39±0.24 | 4.42±0.23 | 4.90±0.28 | 5.50±1.05 |
| Na (mmol/L) | 148.9±1.0 | 149.2±0.5 | 148.3±1.4 | 147.9±1.0 |
| Cl (mmol/L) | 114.1±2.3 | 114.0±1.5 | 113.4±2.8 | 115.1±3.3 |

Suppl. Table 3 Effects on organ weights in experimental rats for RFPH at the terminal stage（g, mean ± s, n = 10）

|  | Parameter | Negative control group | 0.625 g/kg BW | 1.25 g/kg BW | 2.50 g/kg BW |
| --- | --- | --- | --- | --- | --- |
| Females | Brain | 1.513±0.117 | 1.486±0.118 | 1.428±0.093 | 1.488±0.084 |
|  | Heart | 1.080±0.098 | 1.093±0.106 | 1.075±0.088 | 1.141±0.084 |
|  | Liver | 9.00±1.44 | 8.77±0.98 | 9.34±0.73 | 9.49±1.17 |
|  | Kidneys | 2.17±0.31 | 2.19±0.26 | 2.31±0.20 | 2.37±0.07 |
|  | Spleen | 0.598±0.046 | 0.545±0.061 | 0.579±0.099 | 0.611±0.050 |
|  | Uterus | 0.74±0.36 | 0.70±0.22 | 0.69±0.18 | 0.74±0.21 |
|  | Ovaries | 0.198±0.034 | 0.173±0.021 | 0.177±0.017 | 0.189±0.050 |
|  | Thymus | 0.367±0.071 | 0.364±0.068 | 0.360±0.084 | 0.399±0.083 |
|  | Adrenals | 0.101±0.014 | 0.089±0.022 | 0.084±0.033 | 0.098±0.034 |
|  | Fasting BW | 304.8±22.8 | 305.0±25.5 | 302.6±17.8 | 304.2±32.7 |
| Males | Brain | 1.587±0.046 | 1.625±0.102 | 1.651±0.118 | 1.614±0.123 |
|  | Heart | 1.627±0.150 | 1.794±0.211 | 1.746±0.236 | 1.674±0.202 |
|  | Liver | 15.00±0.76 | 15.24±1.82 | 16.14±1.68 | 15.77±2.44 |
|  | Kidneys | 3.34±0.25 | 3.60±0.40 | 3.60±0.30 | 3.43±0.27 |
|  | Spleen | 0.837±0.107 | 0.841±0.119 | 0.843±0.208 | 0.868±0.168 |
|  | Testes | 3.682±0.251 | 3.664±0.274 | 3.680±0.292 | 3.598±0.216 |
|  | epididymides | 1.46±0.11 | 1.41±0.09 | 1.56±0.20 | 1.52±0.11 |
|  | Thymus | 0.444±0.099 | 0.440±0.079 | 0.439±0.100 | 0.464±0.114 |
|  | Adrenals | 0.094±0.030 | 0.092±0.032 | 0.081±0.024 | 0.095±0.034 |
|  | Fasting BW | 495.3±22.7 | 496.4±36.0 | 498.6±25.6 | 498.4±33.7 |

Suppl. Table 4 Effects on organ-to-body weight ratio in experimental rats for RFPH at the terminal stage (%, mean ± s, n = 10 ）

|  | Parameter | Negative control group | 0.625 g/kg BW | 1.25 g/kg BW | 2.50 g/kg BW |
| --- | --- | --- | --- | --- | --- |
| Females | Brain (%) | 0.50±0.04 | 0.49±0.06 | 0.47±0.04 | 0.49±0.04 |
|  | Heart (%) | 0.356±0.042 | 0.360±0.040 | 0.356±0.031 | 0.379±0.045 |
|  | Liver (%) | 2.94±0.31 | 2.88±0.28 | 3.09±0.21 | 3.15±0.50 |
|  | Kidneys (%) | 0.71±0.07 | 0.72±0.09 | 0.76±0.05 | 0.79±0.11 |
|  | Spleen (%) | 0.24±0.13 | 0.23±0.07 | 0.23±0.07 | 0.25±0.08 |
|  | Uteruses (%) | 0.197±0.020 | 0.180±0.025 | 0.190±0.023 | 0.203±0.031 |
|  | Ovaries (%) | 0.066±0.015 | 0.057±0.008 | 0.059±0.007 | 0.063±0.018 |
|  | Thymus (%) | 0.121±0.025 | 0.120±0.023 | 0.119±0.025 | 0.133±0.031 |
|  | Adrenals (%) | 0.033±0.006 | 0.029±0.007 | 0.028±0.011 | 0.033±0.012 |
| Males | Brain (%) | 0.32±0.02 | 0.33±0.02 | 0.33±0.03 | 0.32±0.02 |
|  | Heart (%) | 0.330±0.040 | 0.361±0.033 | 0.350±0.046 | 0.337±0.041 |
|  | Liver (%) | 3.03±0.21 | 3.07±0.29 | 3.24±0.34 | 3.17±0.46 |
|  | Kidneys (%) | 0.68±0.06 | 0.72±0.07 | 0.72±0.07 | 0.69±0.08 |
|  | Spleen (%) | 0.75±0.07 | 0.74±0.07 | 0.74±0.08 | 0.72±0.05 |
|  | Testes (%) | 0.29±0.03 | 0.28±0.02 | 0.31±0.05 | 0.31±0.03 |
|  | epididymides (%) | 0.170±0.025 | 0.169±0.022 | 0.170±0.043 | 0.173±0.026 |
|  | Thymus (%) | 0.090±0.021 | 0.090±0.020 | 0.088±0.018 | 0.093±0.023 |
|  | Adrenals (%) | 0.019±0.006 | 0.019±0.007 | 0.016±0.004 | 0.019±0.007 |

Suppl. Table 5 Effects of RFPH on urine test results in experimental rats at the medium stage (n=5)

| Parameter | Degree | Females | | Males | |
| --- | --- | --- | --- | --- | --- |
|  |  | Satellite control group | Satellite high-dose group | Satellite control group | Satellite high-dose group |
| Glu | - | 5 | 5 | 5 | 5 |
|  | ± | 0 | 0 | 0 | 0 |
| PRO | - | 2 | 3 | 2 | 1 |
|  | ± | 3 | 1 | 2 | 3 |
|  | 1+ | 0 | 1 | 1 | 1 |
| BLD | - | 4 | 4 | 5 | 5 |
|  | ± | 1 | 1 | 0 | 0 |
| SG | 1.005 | 2 | 2 | 0 | 0 |
|  | 1.010 | 1 | 1 | 0 | 1 |
|  | 1.015 | 2 | 2 | 4 | 4 |
|  | 1.020 | 0 | 0 | 1 | 0 |
| pH | 6.5 | 0 | 0 | 1 | 0 |
|  | 7.0 | 0 | 0 | 2 | 3 |
|  | 7.5 | 3 | 2 | 1 | 1 |
|  | 8.0 | 1 | 1 | 1 | 1 |
|  | 8.5 | 1 | 2 | 0 | 0 |

Glu, Urine glucose; PRO, urine protein; BLD, urine occult blood; SG, urine specific gravity; pH, Urine pH.

Suppl. Table 6 Effects on body weight in pregnant rats

| Group | Number of fertilized rats | Number of pregnant rats | Body weight (g, mean ± SD) | | | | | | Weight gain (g) | Net weight gain (g) |
| --- | --- | --- | --- | --- | --- | --- | --- | --- | --- | --- |
|  |  |  | 0 day | 6 day | 9 day | 12 day | 15 day | 20 day |  |  |
| Control group | 19 | 17 | 245.6±16.5 | 284.7±18.8 | 303.4±25.4 | 328±17.0 | 348.3±16.6 | 425.8±20.9 | 141.1±17.0 | 54.7±11.0 |
| 1.25 g/kg BW | 18 | 16 | 242.9±11.3 | 279.9±12.9 | 305.4±17.4 | 324.2±14.5 | 341.6±26.7 | 430.4±22.9 | 150.5±17.5 | 60.3±16.9 |
| 2.5 g/kg BW | 19 | 19 | 242.9±12.7 | 282.8±14.1 | 303.0±15.3 | 321.8±15.7 | 339.9±25.7 | 431.3±22.1 | 148.6±16.8 | 55.0±14.2 |
| 5.0 g/kg BW | 19 | 17 | 241.2±13.5 | 278.8±20.4 | 299.9±17.7 | 321.3±22.8 | 339.9±27.7 | 423.5±33.0 | 144.7±24.5 | 60.0±12.9 |
